#  Longitudinal assessment of health-span and pre-death morbidity in wild type *Drosophila*

**DOI:** 10.18632/aging.101880

**Published:** 2019-03-27

**Authors:** Alexandros Gaitanidis, Agapi Dimitriadou, Harold Dowse, Subhabrata Sanyal, Carsten Duch, Christos Consoulas

**Affiliations:** 1 Laboratory of Experimental Physiology, National and Kapodistrian University of Athens, Athens, Greece; 2 Department of Mathematics and Statistics, University of Maine, Orono, ME 04469, USA; 3 Cell Biology Department, School of Medicine, Emory University, Atlanta, GA 30322, USA; 4 Institute of Developmental Biology and Neurobiology, Johannes Gutenberg University Mainz, Mainz, Germany

**Keywords:** aging, demography, Drosophila, motor behavior, diet, invertebrate disability

## Abstract

The increase in human life expectancy is accompanied by age-related cognitive and motor disability, thus raising the demand for strategies toward healthy aging. This requires understanding the biology of normal aging and late-life functional phenotypes. Genetic model organisms, such as *Drosophila melanogaster*, can help identifying evolutionary conserved mechanisms underlying aging. Longitudinal assessment of motor performance of more than 1000 individual flies revealed age-related motor performance decline and specific late-life motor disabilities. This allows defining heath- and ill-span and scoring late-life quality of individual flies. As in mammals, including humans, onset, duration, severity, and progression dynamics of decline are heterogenic and characterized by both, progressive worsening and sudden late-life events. Flies either become increasingly incapacitated by accumulating disability over multiple days prior to death, or they escape disability until few hours prior to death. Both late-life trajectories converge into a terminal stage characterized by stereotypical signs of functional collapse and death within 3 hours. *Drosophila* can now be used to evaluate life prolonging manipulations in the context of late-life quality. High sugar diet increases lifespan and late-life quality, whereas lifespan prolonging antioxidant supplementation has either no, or negative effects on late-life quality, depending on base diet and gender.

## Introduction

Human life expectancy has markedly increased throughout the world. However, the flip side of the coin is an increased prevalence of impairments in elderly people [1,2]. This can significantly lower life quality and represents a substantial burden for health care systems [3,4]. A fundamental prerequisite toward developing future strategies for healthy aging is to understand the biology and the demography of normal late-life pathophysiology.

Animal models, including invertebrates such as the nematode worm, *C. elegans,* and the fruit fly, *Drosophila melanogaster*, have helped identifying conserved molecular mechanisms underlying aging [5,6]. In *C. elegans*, for example, health-span is defined as the period of robust spontaneous body movements that precede age-related decline. Disability is divided into mid- and late-life disability associated with reduced spontaneous movements, followed by a period of frailty when spontaneous activity ceases [7]. In insulin/insulin-like signaling (IIS) pathway mutants lifespan and mid-life disability are extended, but the frailty period remains unaltered [8]. Insulin signaling is also a major factor of aging in mammals [9,10] and in fruit flies [11], thus indicating that some mechanisms of aging are highly conserved.

In *Drosophila* numerous genetic, pharmacological, and dietary manipulations have been reported to prolong lifespan [11] and/or to postpone age-related motor [12] and cognitive decline [13]. In some cases the *Drosophila* system has allowed to begin pinpointing the underlying molecular and cellular mechanisms [14]. However, the demography of *Drosophila* late-life pathophysiology, and thus, also the effects of life-prolonging interventions on late-life quality remain largely unknown.

Toward addressing this issue we have characterized onset and progression dynamics of *Drosophila* age-dependent motor pathophysiology in longitudinal studies. We found age-related decline in motor performance and characterized specific late-life motor disabilities. Flies either escape disability until few hours prior to death (wellderlies/escapers) and die rapidly, or disability may be manifested and accumulating for days prior to death (illderlies/delayer). Both, escapers and delayers exhibit stereotypic patterns of functional collapse during the last ~3 hours prior to death. Moreover, late-life quality is highly variable because onset, duration, severity, and progression dynamics of decline are heterogenic, largely independent of age at death, and characterized by both, progressive worsening and sudden late-life events. Therefore, *Drosophila* late-life motor pathophysiology bears similarities to what has been reported for humans (see discussion).

We next used our characterization of *Drosophila* late-life pathophysiology to assess the effects of lifespan extending dietary manipulations on late-life quality. High sugar diet increases lifespan and late-life quality [15–17]. By contrast, supplementation with antioxidants prolongs lifespan but has no beneficial, or even negative effects on late-life quality, depending on base diet and gender. Therefore, *Drosophila* can now be used to evaluate life prolonging manipulations in the context of life quality.

## RESULTS

### Late-life motor pathology in *Drosophila*

**Figure 1:**
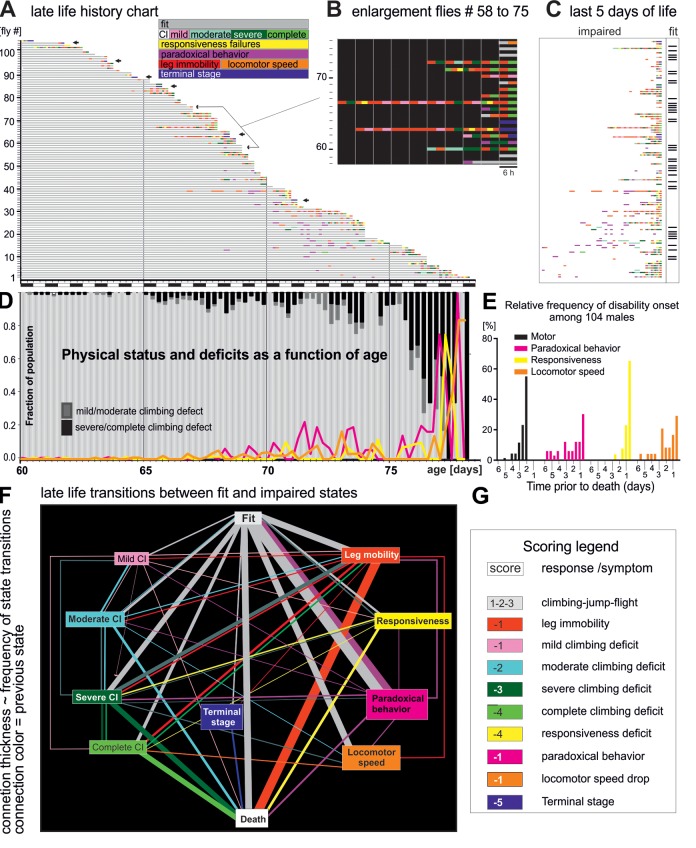
**Late-life pathophysiology of locomotor behavior.** (**A**) Life history chart for 104 flies individually tested in the startle assay every 6 hours from the age of 60 days until death. Gray bars indicate health-span and colored bars disabilities of different categories (see colored inset, note that flies can also receive multiple scores at one testing time, e.g. leg immobility and climbing deficit). Arrows mark individuals that exhibited hyperactivity prior to death (see also Figure 3). Black and white bars on x-axis indicate day-night cycle. (**B**) Selective enlargement of the last 60 hours of life of flies 58 to 75. (**C**) Time enlargement of the last 5 days for all flies. (**D**) The fraction of the cohort being either fit (light gray bars) or showing climbing impairments (mild/moderate dark gray bars; severe/complete, black bars) plotted over time. The percentages of animals with impaired responsiveness (yellow), reduced moving speed (orange), and paradoxical behavior (purple) are depicted by colored lines. (**E**) Frequency distribution of the onset of four disability categories for all 104 flies. (**F**) Ethogram depicts the relative frequencies of transitions (line thicknesses) between fit (grey box), various pre-death impairments (colored boxes), and death (white box). The thickness of the connecting lines depicts the relative frequency of the occurrence of the respective state transition, and the color indicates the previous state. (**G**) Scores used for quantification of the degree of fitness.

**Figure 2:**
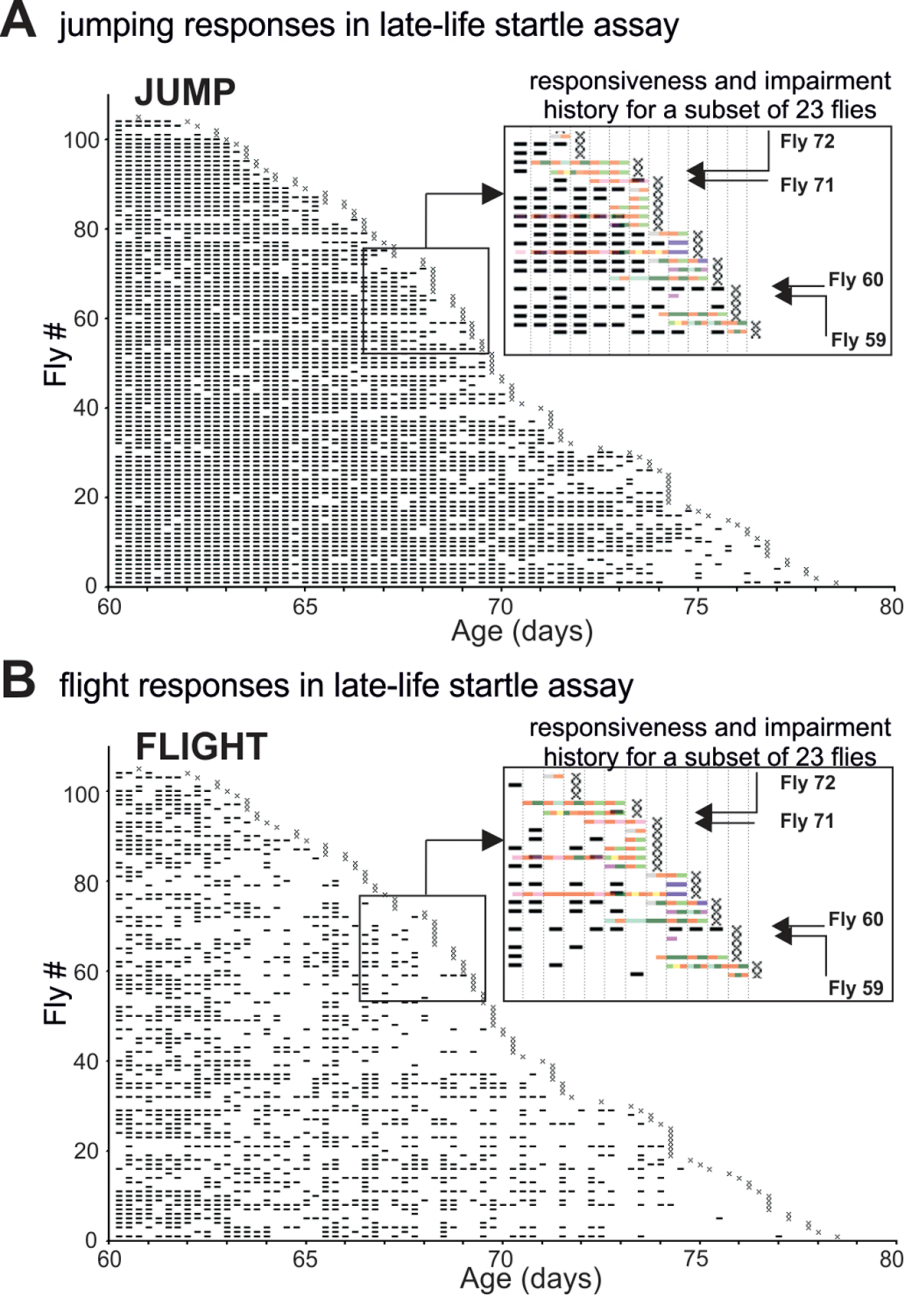
**The pathophysiology of motor performance in the startle assay.** (**A-B**) Event-history charts depict jump (**A**) and flight (**B**) responses of 104 males tested individually every 6 hours between 60d of age and death (see also Figure 1 A). For each fly and each trial responsiveness is indicated by a dash and irresponsiveness by a blank. Insets show overlays of the impairment history (see Figure 1A-C) and the jump/flight responses for a subset of individuals. Note that impairment span was accompanied by a significant reduction in the numbers of jump or flight responses. Four individual flies (F59, F60, F71, F72) were selected as representative case-reports. Fly 59 exhibited paradoxical behavior 18h prior to death and showed no jumping or flight responses during the last 60h of life. Fly 60 showed no impairment and responded with jumping or flight at almost every single trial. Fly 71 was diagnosed with mild climbing impairment and left femur-tibia joint immobility 18 h prior to death. It performed jump but no flight responses. Fly 72 had a defective prothoracic femur-tibia joint and showed multiple climbing defects already 30 hours prior to death. It failed to respond at every late-life trial.

We analyzed late-life motor pathophysiology during normal aging of flies. To account for heterogeneity among individuals we conducted longitudinal motor testing. A motor fitness biomarker in flies is robust escape in response to life-threatening stimuli [18], which can be tested by the “startle assay”. Flies reared in food containing vials respond to mechanical stimulation by either (*a)* climbing towards the top of the vial, (*b)* jumping, or (*c)* performing a short flight (see methods). In the first experimental run, from a total of 224 Oregon-R males (five replicates), we singled out 104 healthy flies at the age of 60 days and tested each fly every 6 hours from day 60 of age until death (Figure 1A, Supplementary Figure 1A, D). In the same experimental run, another population of single males (N=69) was not tested but scored for survival (control). Experimental testing every 6 hours did not reduce lifespan (Supplementary Figure 1D). An event history chart summarizes health-span (Figure 1A, gray bars), impairment-span (Figure 1A-C, colored bars), and lifespan for all 104 flies between the age of 60 days and death (Figure 1A). Startle response impairments were detected in 64% of all individuals and are color coded for impairment type (see inset Figure 1A and below). The remaining 36% were devoid of pathological phenotypes (Figure 1A) and responded normally to the stimulation by climbing, jumping, or flying, even 6 hours prior to death (Figure 2A, B). We refer to flies without any motor impairment until 6 hours prior to death as “wellderlies”.

To characterize onset and progression dynamics of age-dependent decline, pre-death motor impairments were categorized. Category 1 signifies climbing impairments and is sub-categorized by severity (color coded in Figure 1A, see also Table 1). Category 2 demarks a lack of responsiveness to the stimulation (Figure 1A, yellow bars). Category 3 (Figure 1A, magenta bars) denotes a failure to respond with goal directed behavior (instead of a normal escape response, the animal shows e.g. intense walking in circles, or fencing against an imaginary opponent, Movie 1). We refer to this as “paradoxical” motor responses. Category 4 (Figure 1A, red bars) represents leg mobility impairment (Movie 2). This type of impairment was found mostly in femur-tibia joints of pro- and metathoracic legs. Interestingly, electrical responses of leg muscles upon motoneuron stimulation were normal in animals with immobilized legs (Supplementary Figure 2), suggesting mechanical defects in joint articulation or defective electro-mechanical coupling in muscle. Category 5 signifies a substantial decrease in locomotion speed (Figure 1A, orange bars), and category 6 refers to morbid animals in a supine posture (Movie 3), which occurs 2-3 hours prior to death (Figure 1A, blue bars, terminal stage).

**Table 1:** Categories and scores of age-dependent pathophysiology.

**physical status / impairment**	**description**	**score**
fit	normal escape responses. Flies respond with climbing (1), jumping (2), or flying (3).	1-3
leg joint immobility	leg joint defect (leg permanently extended or retracted). Defect is persistent	-1
mild climbing deficit	fly displays mild climbing defect, such as slow speed and defect in a leg but can climb to the top	-1
moderate climbing deficit	fly climbs no higher than up to 1/3 of the vial’s height.	-2
severe climbing deficit	fly can barely climb (few mm) before falling off the wall on its back. Defect is persistent.	-3
complete climbing deficit	fly cannot climb the wall of the vial. Defect is persistent.	-4
responsiveness deficit	absent startle response, extremely week reaction to stimulation	-4
terminal stage	fly is morbid. Defect is persistent and is followed by death.	-5

Until age 65 days only few flies showed impairments (Figure 1A, D). This fraction increased between age 65 days and age 75 days, when climbing (mild/moderate, severe/complete) or other disabilities became present in about 20% of the flies. On average, impairments appeared during the last 4-6 days or 8% of life. Within the last 48 hours of life, the majority of flies (> 80%) exhibited locomotor disabilities (e.g. dragging a leg, or failure to climb), or showed very weak escape responses (Figure 1E). By contrast paradoxical behavior and locomotor speed decline may occur earlier, already within the last 6 days of life (Figure 1E). We define ill-span as the continuous morbid period at the end of lifespan that begins with the appearance of climbing and responsiveness disabilities until death. Since paradoxical behavior occurs transiently in fit individuals, health-span includes cases of fit individuals exhibiting this behavior.

For comprehensive visualization all physical state transitions were plotted as ethogram (Figure 1F). The thickness of each state-connecting line represents the percentage of flies showing that transition, and line colors demark the state prior to a transition. One third of all flies died without any impairment until the last trial before death (vertical “fit to death” transition). The most common transitions from fitness to pre-death impairments were (i) to paradoxical behavior (magenta box, reversible), (ii) leg immobility (red box, irreversible), (iii) locomotor speed (orange box, irreversible) and (iv) severe/complete climbing impairments (green boxes). Severe and complete climbing inabilities were usually not the end-product of gradually worsening mild or moderate defects, but mostly occurred suddenly and were almost always persistent. By contrast, milder climbing defects occurred recurrently. Paradoxical behavior occurred sporadically and rarely directly preceded another impairment or death.

In sum, this assay indicated that flies can exhibit two modes of transitioning to death, either they die without any late-life motor impairments until 6 hours prior to death, or they show pre-death impairments of substantial heterogeneity and diversity. To confirm that these are general features of wild type *Drosophila*, and not a peculiarity of one laboratory strain, we conducted a second experimental run with 48 Oregon-R males (N=92, 12 censored, two replicates; Supplementary Figure 1B). Although this run was performed a year later with Oregon-R flies newly obtained from the Bloomington stock center, it revealed no significant differences in ill-span durations and survival trajectories (Supplementary Figure 1B, C, D). In addition, we repeated the experiment also with a second wildtype strain, Lausanne-S, and obtained similar results (see below, Figure 5).

For further quantification each behavioral category was assigned a score (Figure 1G, see also Table 1). Healthy motor responses to stimulation were assigned positive values (1 for climbing, 2 for jumping, 3 for flight), whereas impairments were assigned with negative values reflecting the severity of the impairment (Figure 1G).

### Motor performance but not the degree of disability decline gradually

**Figure 3:**
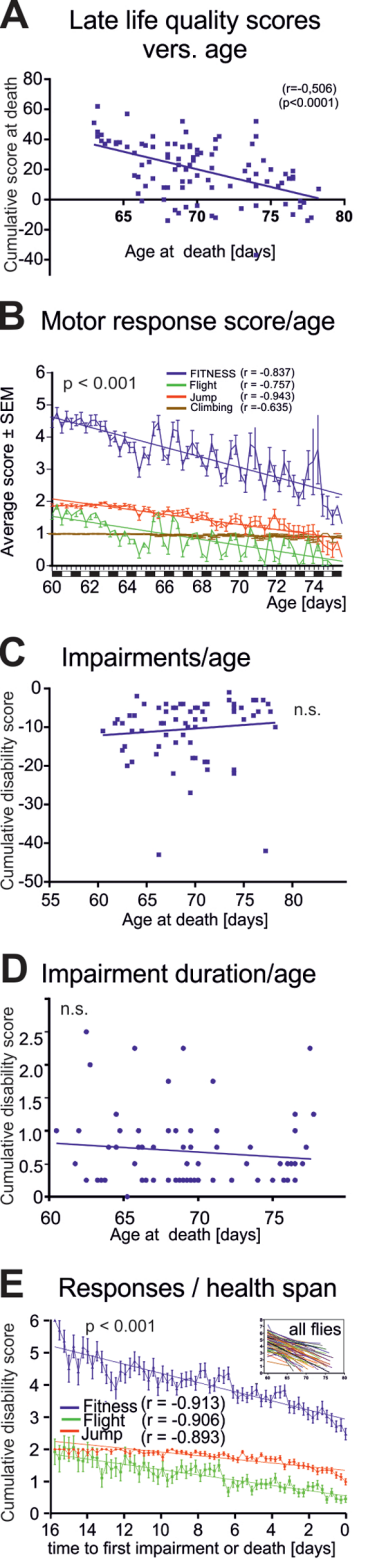
**Characteristics of late-life impairments.** (**A**) Cumulative life quality score during the last three days for each animal decreased significantly with age of death (Pearson’s correlation coefficient = 0.506; p < 0.001). (**B**) Average fitness scores as obtained from the startle assay reveal a marked decline in flight (green line) and jumping (red line) responses and a moderate decrease in climbing responses with age. Overall performance score (blue, sum of all scores) decreased with age. (**C**) Cumulative impairment scores (sum of all negative scores for each animal with disabilities) revealed no correlation between degree of impairments and age at death (Pearson’s correlation coefficient = 0.016; p > 0.2). (**D**) Impairment duration did not correlate with age at death (Pearson’s correlation coefficient = -0.011; p > 0.2). (**E**) Cohort fitness scores decreased significantly during health-span (we started scoring healthy flies at 60 days of age. Note that the oldest fly that did not show any impairment until death was 76 day old, thus resulting in a 16 days observation period, from 60-to-76 days, see x-axis in E) (Pearson’s correlation coefficient = -0.913; p < 0.001).

Overall late-life quality was assessed for each fly by a cumulative score during the last three days of life. Late-life quality showed a significant negative correlation to the age at death (Figure 3A, p < 0.001, Pearson r test). This could either be caused by a decline in the intensity of healthy motor responses, or by cumulating disabilities, or both. Separating healthy motor performance (positive scores) from impairments (negative scores) revealed a significant decline in motor performance intensity with age (Figure 3B, blue line). Older flies responded less often with jumping or flying (Figure 3B, green line flight, red line jump), whereas the frequency of climbing responses remained more stable until death (Figure 3B, brown line). The fluctuations along the steadily declining performance scores with age were due to the light/dark cycle (Figure 3B). By contrast, neither disability scores reflecting the severity of motor defects (Figure 3C, r = 0.1021 p = 0.4002, Pearson test), nor the duration of ill-span (Figure 3D, r = -0.011, p>0.2, Pearson test) increased significantly with age at death.

Therefore, at the cohort level, longevity was not accompanied by cumulating motor and other disabilities, but instead, by decreased escape response performance intensity (more climbing versus jumping or flying responses) that started already during health-span (Figure 3E).

### Silent death with accumulating motor disabilities versus sudden death of wellderlies

**Figure 4:**
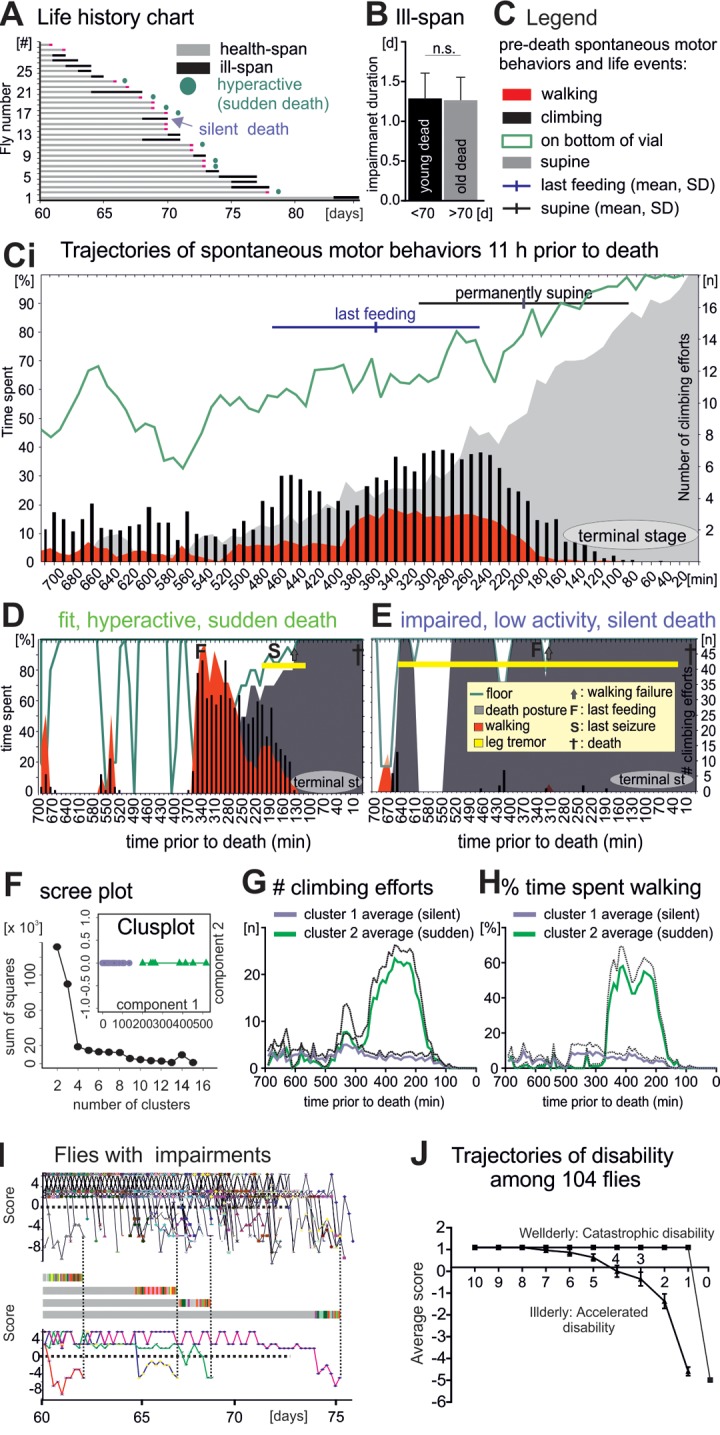
**Flies die in two different modes: Sudden versus silent death.** (**A**) Life history chart for 30 flies filmed continuously from age 60 days until death. Health-span is indicated by horizontal gray bars and ill-span by black bars. Circles mark animals without impairments but with a short period of hyperactivity during late-life (see below). Black arrows demarks an example animal with multiple days of ill-span. (**B**) Impairment duration was statistically similar in flies dying younger (black bar) versus older than 70 days (grey bar, students T-test, p > 0.9). (**C**) Legend for (**Ci**), which shows the occurrence of different spontaneous behaviors during the last 11 hours of life plotted as cohort averages in time bins of 10 minutes (x- axis). Percent time spent (left y-axis) walking (red), standing on the wall or the bottom of the vial (green line), being immobile or in a supine position (gray). Black bars depict the number of climbing efforts (right y-axis). Horizontal bars depict cohort averages and SEMs for the time of the last feeding (blue) and for entering a permanent supine position (black). (**D-E**) Same data presentation as in C but for a representative animal with high locomotor activity and no impairments until the last day (**D,** sudden death) as compared to a representative animal with impairments and low locomotor activity (**E,** silent death). (**F**) Principle component analysis revealed two distinct clusters of animals. (**G, H**) Number of climbing efforts (**G**) and time spent walking (**H**) (averages, solid lines; SD, dotted lines) in bins of 10 minutes during the last 11 hours of live for all cluster 1 animals (blue) versus all cluster 2 animals (green). (**I**) Fitness scores of every illderly fly from the startle assay cohort of 104 flies (see also Figure 1) plotted over age (upper panel). Fitness to impairment state transition can be sudden or gradual as exemplified by the score changes of four individuals (middle and lower panels). (**H**) The average score of all wellderly versus all illderly flies from the startle assay (see figure 1) plotted over the last 10 days of life follows different trajectories.

Our longitudinal study revealed that about 35% of all flies died without late-life impairments. However, since the temporal resolution during this assessment was 6 hours, we might have missed distinct patterns of organismal collapse prior to death. Therefore, we continuously video captured spontaneous behavior of an additional cohort of 30 individual flies from the age of 60 days until death. Death was called when a fly was completely immobile, laid on its back (supine [19,20]), and showed no responses to visual or tactile stimulation. All flies entered the experiment without any obvious motor impairment. As in the “startle assay” (Figure 1) about 60% of the animals showed signs of irreversible disabilities in spontaneous motor behavior for more than one day during late-life (Figure 4A). Conversely, 40% of all individuals showed no motor disabilities until 11 hours before death (Figure 4A). Again, severity and duration of motor impairments depended not on age at death (Figure 4B), a feature also reported for human aging [1].

To characterize the pathophysiology of dying we analyzed the last 11 hours prior to death in bins of 10 minutes. Relative to average total lifespan this compares to about the last 6 months of human life assessed every 2 to 3 days. During their last 11h flies spent increasingly more time at the bottom as compared to the wall of the vial (Figure 4C, Ci, green line). This was accompanied by the cessation of feeding, and surprisingly, by an increase in average locomotor activity at the population level (Figure 4Ci). Finally, about 2-3 hours prior to death all flies became largely immobile, showed periods of leg tremor (Movie 3), rhythmic abdominal bending, proboscis extension/retraction movements, and occupied a supine posture (supine Figure 4C, Ci, gray area). No fly ever escaped from this condition, and thus, we named it terminal stage of life. After death a rigor mortis-like state was characterized by permanent abdominal bending and leg retraction.

As stated above, on the cohort level this characteristic sequence of pre-death deterioration was accompanied by an intermittent increase in spontaneous locomotor behavior (Figure 4Ci, red area, walking; black bars, climbing). Analysis of each individual indicated that some flies displayed hyperactivity for about 2 hours (for an example see Figure 4D, for a visual separation of phenotypes see Supplementary Figure 3), whereas others continuously displayed low locomotor activity (for an example see Figure 4E, Supplementary Figure 3). Principal component analysis revealed that most of the variability in pre-death activity measurements could be explained by two clusters (Figure 4F). Cluster 1 animals showed no increase in average climbing (Figure 4G) or walking activity (Figure 4H), whereas locomotor activity of cluster 2 animals was markedly increased during a sharp time window of 2 hours prior to functional collapse (Figure 4G, H). This indicated two distinctly different pre-death pathophysiologies.

Further analysis revealed that all animals with motor impairments prior to the last day of life (Figure 4A, black bars) died without a phase of increased locomotion (referred to as silent death, Figure 4A, blue arrow). By contrast, 50% of all animals without impairment exhibited pre-death hyperactivity (Figure 4A, green circles, referred to as sudden death). Therefore, the mode of dying (sudden versus silent) depended on the fitness during late-life. Arguably, animals with motor disabilities may simply not be able to display pre-death hyperactivity. However, hyperactivity of old flies without obvious motor disabilities was likely also a consequence of deterioration, because every single fly that we observed in this state died within few hours (n = 40).

In summary, flies died either silently with progressively accumulating disabilities, or they aged mostly healthy, showed a short period of increased locomotor activity, and then died suddenly. To test for generality, we also analyzed the progression dynamics of ill-span in our longitudinal startle assay study with 104 flies (Figure 1). Plotting the motor performance score of every fly with disabilities over age (Figure 4I) revealed that “fit to impaired” transitions can either be sudden or gradual, as exemplified by score changes of four selected individual flies (Figure 4I, middle and lower panels). Moreover, plotting the average score changes of flies with and without disabilities separately (Figure 4J) revealed two distinct trajectories: First, illderly flies started showing disabilities up to 7 days before death, and disability score worsened exponentially until death (Figure 4J, silent death, accelerating disability). Second, wellderly flies showed no disabilities up to the last day of life, and then showed a steep physical status decline within few hours (sudden death with catastrophic disability). Therefore, at the cohort level, average physical status declines with age, but onset, duration, and severity of age-dependent impairments are variable. The progression of impairment conditions is characterized by both, accumulating disabilities and sudden late-life events.

### Gender and strain dependent differences in lifespan do not affect pre-death pathology

**Figure 5:**
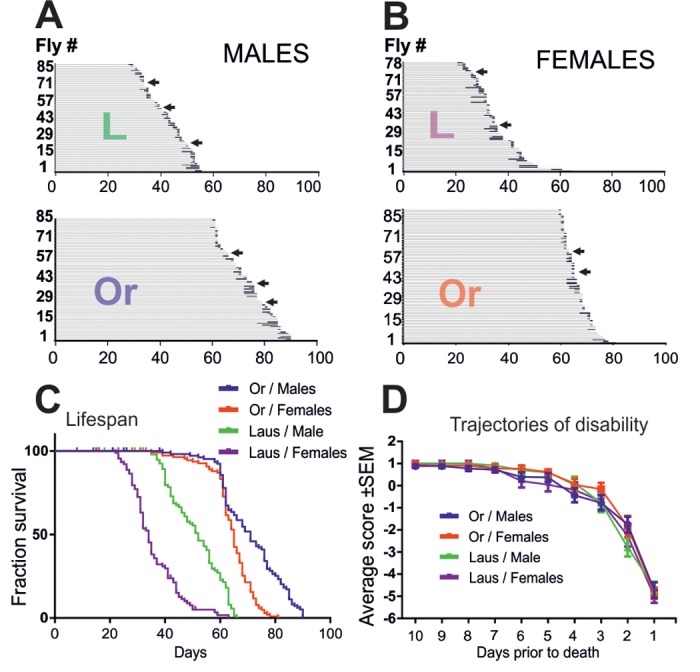
**Oregon-R and Lausanne-S strains display similar late-life pathophysiology.** (**A, B**) Life history chart of Oregon-R and Lausanne-S male (**A**) and female (**B**) flies individually tested in the startle assay daily from the age of 60 days (Oregon-R) and 20 days (Lausanne-S) until death. Gray bars indicate health-span and black bars ill-span. Arrows mark individuals that exhibited hyperactivity prior to death. (**C**) Survivorship curves of male and female Oregon-R and Lausanne-S flies of **A** and **B**. (**D**) Trajectories of disability are plotted separately for Oregon-R and Lausanne-S males and females as average performance scores during the last 10 days prior to death.

In many species, including *Drosophila*, sex and genetic differences have marked influences on lifespan and late-life quality. To examine whether the above described late-life pathophysiology of *Drosophila* differed between different wildtype strains, we repeated longitudinal testing of the physical status with Lausanne-S flies, and in parallel with another cohort of Oregon-R flies. Like previously (Figure 1), physical status was examined at the individual level, but with testing once per day instead of every 6 hours. Since Lausanne-S flies have a shorter lifespan, testing was done from age of 20 days until death for the Lausanne-S populations, and from age of 60 days to death for the Oregon-R population, respectively. To test for gender specific differences, we analyzed males and females separately. In both fly strains, males lived longer than females (Figure 5A, B, C, see Table 2 for statistical analysis). In comparison to Oregon-R flies, Lausanne-S flies had a shorter life span (Figure 5A, B, C). However, neither sex differences nor strain differences showed significant effects on the bimodal course of pre-death activity. As found before (Figure 1), some male and female flies of both strains showed no impairments up to the last day of life to then exhibit hyperactivity followed by impaired climbing, seizures, supine position, leg tremor, and death (see arrows in Figure 5A, B). Furthermore, all impairment categories previously described for Oregon-R were confirmed for Oregon-R females and for both genders of the Lausanne-S strain. Despite different lifespans, the duration of motor impairments did not differ between gender and strains, indicating that when the organism drops beyond a functional threshold, an age-independent and time-fixed process drags the organism to death. The only difference we detected was that responsiveness and speed impairments occur earlier in Oregon-R than in Lausanne-S males (Table 3). The two distinct trajectories of disability, catastrophic (wellderly flies) and accelerated (illderly flies), show no differences among gender and strains (Figure 5D). Next we examined to what extend the period and severity of impairments prior to death can be reduced or ameliorated by environmental (dietary) manipulations in both sexes of the Oregon-R strain.

**Table 2:** Lifespan of cohorts raised in different c:p ratio diets with or without supplementation of curcumin (Cur) and Super Fruit (SF) in the larval stage. Days are days of adulthood.

**Gender-** **c/p**	**Med** **days**	**Mean** **days**	**SE** **days**	**Max LS observed** **/Max LS from replicates (days)**	**p-value** **control 1** **control 2**	**No. un-** **censored**	**No.** **censored**	**No.** **cohorts**	**N**
M-2/1 (con1)	71.0	70.86	1.108	90 (90,90,90,86,86)	(1) -	102	7	5	109
M-2/1+SF	86.0	84.57	1.597	108 (107,107,107,106,108)	< 0.0001	101	9	5	110
M-2/1+Cur	86.0	83.34	1.740	127 (105,106,112,127)	< 0.0001	79	8	4	87
M-8/1 (con2)	88.0	82.20	1.377	101 (101,97,94,97)	< 0.0001 (2) -	102	11	4	113
M-8/1+SF	88.0	81.59	1.500	98 (98,95,95,94)	< 0.0001 0.5035	87	12	4	99
M-8/1+Cur	83.0	81,57	1.119	101 (94,101,101,99,92)	< 0.0001 0.1244	97	7	5	104
M-2/1 Lausanne	52.0	51.03	0.9889	66 (65,66,65,65)	< 0.0001	90	8	4	98
F-2/1	65	64.16	0.7678	81 (77,81,78,72,69)	(1) -	105	10	5	115
F-2/1+SF	71	71.27	1.398	102 (102,100,89,93)	< 0.0001	79	9	4	88
F-2/1+Cur	67	68.06	1.277	103 (82,82,193,86)	< 0.0001	85	10	4	95
F-8/1 (con2)	86	78.73	1.556	98 (97,97,90,98,94,96)	< 0.0001 (2) -	112	10	6	122
F-8/1+SF	76	76.27	1.022	100 (95,100,88,90,88)	< 0.0001 < 0.0001	109	10	5	119
F-8/1+Cur	77	75.24	1.145	95 (95,88,85,86)	< 0.0001 < 0.0001	85	12	4	97
F-2/1 Lausanne	34	35.25	0.9328	63 (63,58,59,50)	< 0.0001 < 0.0001	83	10	4	93

Maximum lifespan was from adult emergence until the last day a fly was alive. The log-rank analysis was used to determine whether lifespan differed between control of Oregon-R and of Lausanne-S flies (gray, fed on c:p 2:1 without supplementation throughout life) and different feeding paradigms. Survival data from all experiments were pooled for the analysis. Median lifespan (Med), mean lifespan (Mean), standard error (SE), and maximum lifespan (Max. LS) are listed. The maximum lifespans of each replicate are listed in brackets..

**Table 3:** Disability duration, irresponsiveness, and motor impairment duration in days in Oregon-R and Lausanne-S male and female flies.

**Gender-** **c/p**	Disability duration (days) (average±SEM)	p-value	Responsivness onset – days (average±SEM)	p-value	Speed imp. onset –days (average±SEM)	p-value
Oregon Males	2.500± 0.2601		3.375± 0.5650		4.103±0.4857	
Lausanne Males	2.169± 0.1795	> 0.9999	1.692± 0.2861	**0.0280**	2.000±0.2646	**0.0078**
Oregon females	1.787± 0.1898		2.150± 0.3185		2.667±0.3318	
Lausanne Females	2.509± 0.2419	0.1361	1.739± 0.2896	> 0.9999	3.074±0.4831	> 0.9999

Bold/highlighted numbers indicate significance. M=Male, F=Female.

### Dietary interventions can prolong lifespan and compress or extend pre-death morbidity

**Figure 6:**
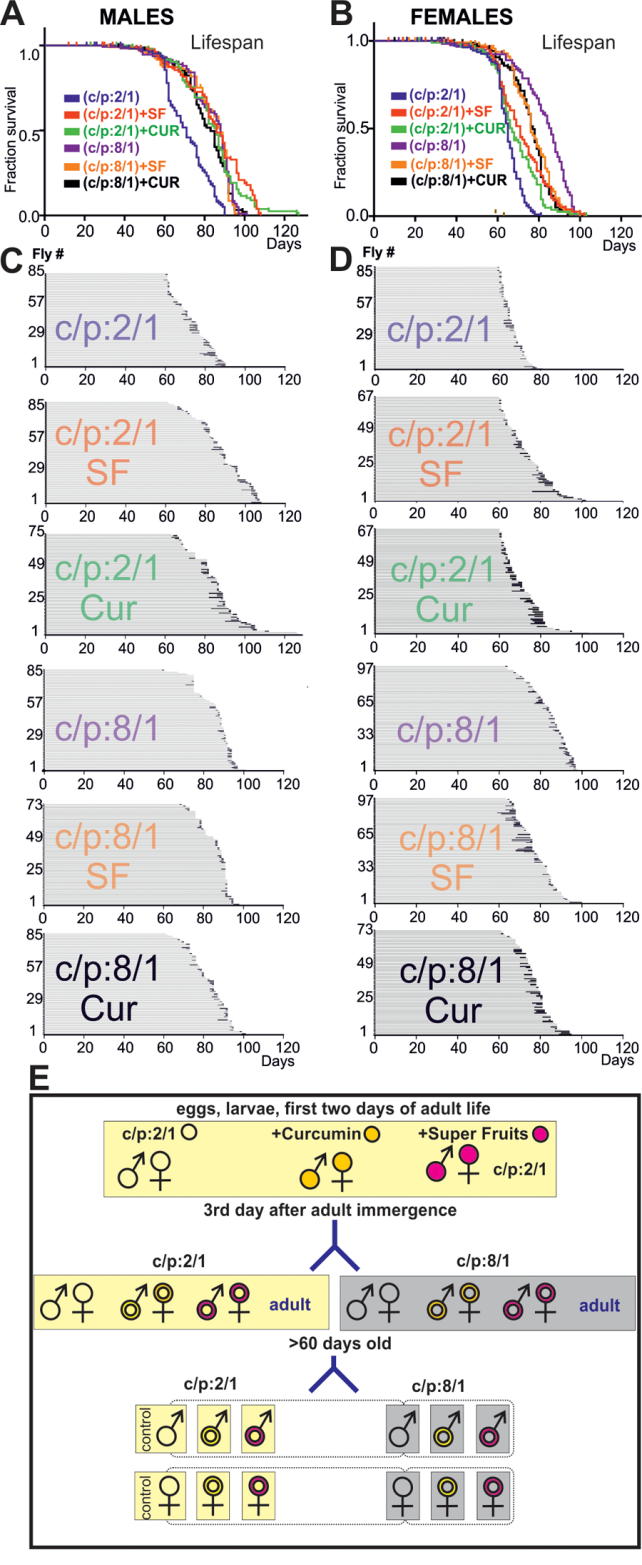
**High sugar diet and antioxidants increase lifespan.** (**A, B**) Survivorship curves of males (**A**) and females (**B**) of six cohorts of flies, each raised on a different diet (C:P 2/1, blue; C:P 2/1 + curcumin, green; C:P 2/1, + super fruits, red, C:P 8/1, purple, C:P 8/1 + curcumin, black; C:P 8/1 + super fruits, orange). For statistics see Table 2. (**C, D**) Life history charts of all six male (**C**) and all six female (**D**) populations. Horizontal gray bars depict health-span and black bars ill-span. (**F**) Schematic representation of the different feeding paradigms used.

Dietary restriction or supplementation can increase lifespan in multiple species, including *Drosophila* [6,13,16,21], but the effects of life prolonging interventions on late-life pathophysiology remain largely unknown. To start addressing this question we raised wildtype flies on different diets. It has been shown that high carbohydrate vs protein concentration ratio increases lifespan [15–17]. Furthermore, feeding larvae or young flies with 100mM curcumin is known to increase longevity in *Drosophila* [22], and the fruit cocktail extract, Super Fruit (SF), supplemented to the fly food, provides strong antioxidant defence [23]. Thus we used two diets: the base diet (carbohydrates/protein ratio 2/1, C/P:2/1) and the base died with high carbohydrates/protein ratio; (C/P:8/1). First, three Oregon-R larvae populations were raised, the first on the base died C/P:2/1 (control population), the second population on the base died supplemented with curcumin and the third population on the base died supplemented with Super Fruit. After adult emergence, the three 3 days old adult populations (control, Curcumin, SF) were separated into two groups. One was continued to be fed on the base 2:1 C:P ratio diet, and the other one was raised on the 8:1 C:P ratio diet (see Material & Methods, for a schematic of the feeding protocols see Figure 6E). The passage of young adults to high C/P ratio diet increased lifespan by ~20% in both genders (Figure 6A, Β, Tables 2, 4). The supplementation with antioxidants had lifespan extending effects that depended on the C:P ratio of the base diet and on gender. Curcumin and SF increased lifespan of flies fed on low C:P ratio diet. By contrast, when animals were fed on a high C:P ratio diet, antioxidants had no additional effect on male lifespan and even decreased the lifespan of females (Figure 6A, B, for statistics see Table 4).

**Table 4:** The Log-rank (Mantel-Cox) test was used to compare survival curves. Italics indicate statistical values of C:P 8/1 + SF and C:P 8/1 + Cur in relation to *C:P 8/1*.

**Diet**	**Males**	**Females**

	**X^2^**	**p-value**	**X^2^**	**p-value**
**C:P 2/1**				
C:P 2/1+ SF	60.10	**< 0.0001**	13.93	**0.0002**
C:P 2/1 + Cur	44.10	**< 0.0001**	15.94	**< 0.0001**
***C:P 8/1***	78.75	**< 0.0001**	149.1	**< 0.0001**
C:P 8/1 + SF	82.23 *0.4073*	**< 0.0001** *0.5233*	106.1 *25.37*	**< 0.0001** ***< 0.0001***
C:P 8/1 + Cur	43.35 *5.530*	**< 0.0001** *0.0187*	83.08 *32.99*	**< 0.0001** ***< 0.0001***

Bold/highlighted numbers indicate significance.

**Figure 7:**
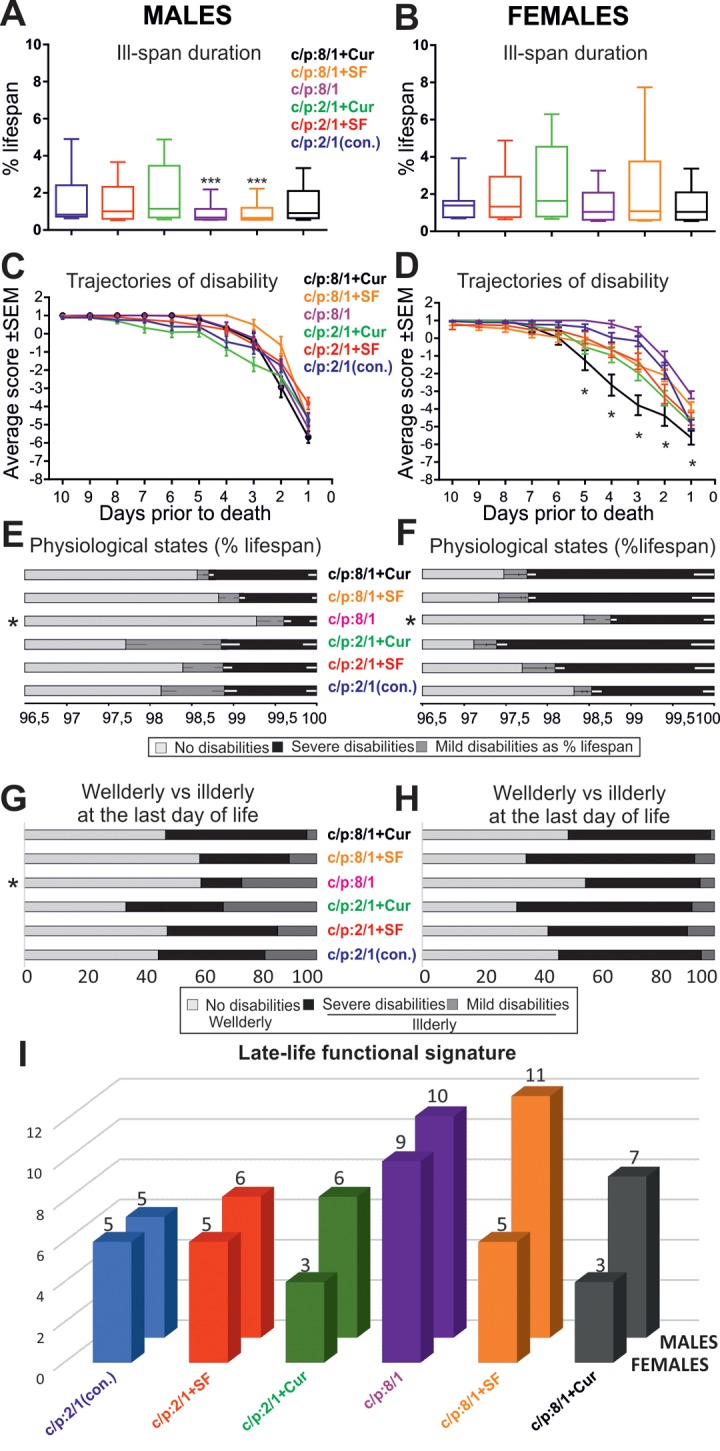
**Dietary interventions can change late-life quality substantially.** (**A, B**) Box plots depict ill-span duration for all 6 male (**A**) and all 6 female (**B**) populations raised on different diets. *** indicates statistical significance with p < 0.001, Kruskal Wallis ANOVA with Dunn’s posthoc testing. (**C, D**) Average disability scores of illderly flies plotted over the last 10 days of life for all 6 male (**C**) and all 6 female (**D**) populations. ** indicates statistical significance (p < 0.01, one way repeated measures ANOVA). **(E, F)** Duration of health-span (light gray bars), mild/moderate ill-span (dark gray bars), and severe/complete ill-span (black bars), as a percentage of lifespan are plotted for each diet and separately for males (**E**) and females (**F**). * indicates statistically significant difference (Kruskal Wallis ANOVA with Dunn’s posthoc testing (**G, H**). The relative percentages of animals without impairments (light gray bars), with mild impairments (medium gray bars) and with severe impairments (dark gray bars) during the last day of life plotted separately for each diet and males (**G**) and females (**H**). * indicates statistically significant differences (p < 0.05, Chi-square test). (**I**) The cumulative startle response score through the last ten days of life (late-life functional signature) plotted for each diet (color coded) and separately for females (front row) and males (back row).

To test whether these dietary interventions affect late-life quality, all flies were housed individually from day 60 until death, and their physical status was examined once per day. At a first glance, ill-span did not change markedly with the food manipulations (see black bars, Figure 6C, D). To determine whether dietary manipulations affected late-life physical parameters we took into account lifespan differences across populations [8]. Quantification revealed that ill-span duration as a percentage of lifespan was similar in all female groups, independent of diet (Figure 7B; Table 4). By contrast, in males it significantly decreased on high sugar diet, with or without the addition of Super Fruit (C:P 8/1, C:P 8/1+SF, Figure 7A, Table 5).

**Table 5:** Disability duration, irresponsiveness, and motor impairment duration as a percentage of lifespan.

Gender- c/p ratio + supplement	Ill-span duration (mean ± SEM)	p-value	Irresponsiveness duration (mean ± SEM)	p-value	Motor imp. duration (mean ± SEM)	p-value
M-2/1 control	1.871± 0.1839		1.1000 ± 0.1113		2.561±0.3224	
M-2/1+SF	1.564± 0.1431	0.5151	0.7547 ± 0.06118	**< 0.0001**	2.643±0.3296	0.7206
M-2/1+Cur	2.267± 0.2575	> 0.9999	1.050±0.1602	**0.0006**	2.711±0.3318	0.6821
M-8/1	1.114± 0.09216	**0.0005**	0.6389±0.03025	**< 0.0001**	0.8393±0.07322	**< 0.0001**
M-8/1+SF	1.069± 0.08322	**0.0009**	0.6852±0.04057	**< 0.0001**	0.7971±0.06551	**< 0.0001**
M-8/1+Cur	1.416± 0.1248	0.3114	0.9914±0.09201	**0.0014**	1.052±0.1069	**< 0.0001**
						
F-2/1 control	1.733± 0.1660		1.235±0.1338		2.302±0.2777	
F-2/1+SF	2.222± 0.2797	> 0.9999	1.379±0.2203	0.0979	2.487±0.3914	0.9817
F-2/1+Cur	2.851± 0.2746	0.1321	1.191±0.1609	0.2578	2.500±0.2996	> 0.9999
F-8/1	1.474± 0.1146	0.1470	0.7029±0.03333	**< 0.0001**	1.278±0.1124	**< 0.0001**
F-8/1+SF	2.611± 0.3216	> 0.9999	0.7320±0.03451	**< 0.0001**	1.439±0.2259	**< 0.0001**
F-8/1+Cur	1.551± 0.1388	0.1840	0.7000±0.03277	**< 0.0001**	4.012±1.558	**< 0.0001**
						

Bold/highlighted numbers indicate significance. M=Male, F=Female.

Motor disability onset was postponed in all male cohorts and in female cohorts raised on high sugar diet, while locomotion speed disability was ameliorated in all cohorts raised on high sugar diet with the exception of females supplemented with curcumin (comparisons to control populations: males C:P 2/1, females C:P 2/1; Table 5).

To test whether impairment progression dynamics change with diet, we plotted the average disability scores for all animals with late-life disabilities (illderlies) for all cohorts (Figure 7C, D). In all male cohorts (Figure 7C) and in most female cohorts (Figure 7D) disability followed the accelerating trajectory also revealed in our longitudinal startle assay study (Figure 4J). By contrast, supplementation of high sugar diet with curcumin significantly increased the speed of functional decay in female flies (Figure 7D, black trajectory, progressive disability). These female flies also exhibited a particular pathology characterized by balloon-like shaped abdomens, a permanently extended proboscis and haemolymph leakage from the leg joints.

We next asked whether disability severity differed between cohorts. In both genders the duration of mild and/or severe disability as a percentage of lifespan was significantly shorter on high sugar diet (see asterisk C:P 8/1, Figure 7E, F). Therefore, high carbohydrate diet prolonged health-span and compressed morbidity. Note that the supplementation of high sugar diet with antioxidants caused no additional improvement, but by contrast, increased the duration of severe motor disability in male and in female cohorts (Figure 7E, F). Moreover, antioxidants had no ameliorating effect on disability severity in animals fed on low carbohydrate diet, and curcumin even prolonged severe disability duration in females on low carbohydrate diet (Figure 7E, F). Disability duration of females was consistently longer than in males, largely independent of diet, longest with antioxidant supplementation, and shorter on high sugar diet (Figure 7E, F).

The relative percentages of healthy (Figure 7G, H, light gray bars), moderately disabled (Figure 7G, H, dark gray bars), and severely disabled (Figure 7G, H, black bars) flies at their last day of life was used as a measure of late-life quality on the cohort level. Relative to total lifespan a day of a fly’s life equals approximately a year of human life. High carbohydrate diet significantly lowered the percentage of disabled males (Figure 7G, Chi-square test, p < 0.01) and had moderately beneficial effects on females (Figure 7H). The addition of antioxidants had either no effect (Figure 7G, males on high carbohydrate plus SF), or it decreased life quality (Figure 7G, H).

Finally, we assessed the effects of diet on overall late-life quality by taking into account the aforementioned five parameters (ill-span duration, responsiveness and speed impairment onset, severe/complete disability duration, percentage of illderly flies at the last day of life). Late-life quality was significantly improved in male and female flies raised on high carbohydrate diet (Figure 7I). Surprisingly, the supplementation of high carbohydrate diet with antioxidants had either no effect (Figure 7I, males) or decreased late- life quality (Figure 7I, females). Similarly, antioxidant supplementation of low sugar diet did not improve late-life quality (Figure 7I).

## DISCUSSION

Despite extensive use of *Drosophila* as a genetic model system in aging research [14,25], the physiological events leading to functional collapse prior to death have remained largely unknown. Our study provides novel demographic data of functional motor decline in elderly flies, including the development of specific late-life motor disabilities and detrimental events prior to death. This now allows assessing physiological age at the level of individual flies, which is useful to pinpoint cellular and molecular causes of functional decline and to study the effects of lifespan increasing manipulations on physical status.

### Characteristics of *Drosophila* late-life motor pathophysiology

Progressive decline in walking ability is one of the most prominent age-related phenotypes in all animals, and it can significantly decrease independence and life quality of elderly humans [26]. Similarly, flies exhibit age-dependent locomotor speed decline [12,27–30]. In accord, we find that motor performance intensity of escape behavior decreases gradually with age on the cohort level, even before specific motor impairments are observed. However, the degree of decline varies markedly between individuals. About 60% of all flies exhibit disabilities for multiple days and become gradually slower between impairment onset and death (referred to as illderlies/delayers). By contrast, in wellderly flies (escapers) climbing ability persists to the last day of life, and climbing speed can remain unchanged between day 60 and the last day of life. These animals even show an intermittent increase in locomotor activity during their last day of life. Therefore, we identified two distinct trajectories of late-life motor decline, one characterized by sudden late-life events and the other one by gradual decline. It has been shown that starved flies display adipokinetic hormone dependent hyperactivity prior to death [31,32]. To test whether hyperactivity as observed during sudden death, was simply caused by a previous cessation of feeding, we tested whether animals undergoing sudden death mode ceased feeding earlier, but we did not find evidence for this hypothesis. Independent of their trajectory of decline, all flies exhibited a stereotypical sequence of functional collapse during the last 2-3 hours of life, characterized by a supine body posture, an inability of righting, leg tremors, and rhythmical proboscis extensions. The cause for this functional collapse remains unknown, but it is a behavioral biomarker of time of death, because no animal ever escapes from this condition and dies within 3 hours. Another reported biomarker of impending death in flies [33], *C. elegans,* and fish (*Danio reiro)* is intestinal barrier dysfunction [34].

Although speculative at this point, one could assume that death occurs upon passing a critical threshold of functional decline. If correct, one might argue that this threshold is lower in wellderly flies, so that they die quickly upon developing disabilities. By contrast, a higher threshold might delay functional collapse and death in illderly flies with accumulating disabilities. Please note that average disability duration of 1.2 days matches an increased average lifespan in illderly flies, thus supporting the idea that functional collapse may be delayed in illderlies. Moreover, ill-span duration is similar in shorter lived Lausanne-S and longer lived Oregon-R flies, as well as following live prolonging manipulations. This contrasts findings in *C. elegans*, where longer lived individuals exhibit a lower rate of physiological decline throughout life but a longer period of declined physiological function prior to death [35].

### Possible mechanistic causes of late-life impairments

We have provided a canvas of pathophysiological traits associated with late-life functional decay. We distinguish four categories of impairments that likely reflect dysregulation in different nervous system parts or in other organs. First, no-goal directed responses to a startle stimulus (paradoxical behavior) likely reflect impairments in higher brain centers, because motor coordination seemed normal in animals responding with fencing or intensive walking in circles to a stimulus that normally elicits escape. By contrast, climbing disabilities likely reflect problems in muscle power so that animals either paused unexpectedly, or even fell off the wall of the vial. Please note that leg coordination was not obviously altered during periods of climbing. A lack of responsiveness to startle stimuli may be caused by sensorimotor malfunction, or by decreased attention or motivation. Finally, locomotor speed reductions are unlikely a consequence of decreased motivation, because speed remained slow even during escape from a life-threating stimulus. Instead, they may reflect metabolic shortfalls. Further analysis will be required to tease apart the cellular causes for these late-life conditions in *Drosophila*. However, having identified specific traits that characterize physiological aging in individual flies now allows comparing molecular and cellular aspects of nervous system function in age-matched animals with and without impairments.

### Drosophila late-life demography reflects some aspects of human late-life demography

Some people reach high ages in good health, while others have poor health and functioning for years prior to death. Disability, a term reflecting difficulties of elderly to perform basic physical activities of daily living, can either be manifested as progressive worsening of physical status, or as a sudden transition from healthy to unhealthy state [3]. Although overall health-span duration is increasing in humans [36], it is not clear whether disability prevalence is increasing [37,38] or decreasing [1,39,40]. Moreover, given the complex pathologies in multiple organ systems, the trajectory of human disability cannot be predicted even if the primary cause ultimately leading to death is known [3]. Thus, functional phenotypes and trajectories of disability are highly heterogeneous and diverse [3,41]. Similarly, we report heterogenic and unpredictable disability onset, disability duration, and physical state transitions on the level of individual fruit flies.

In the *C. elegans* model, disability is defined as a period of reduced locomotor speed during mid- and late-life, followed by a frailty period with the cessation of movement [8,42]. As in humans and in *Drosophila*, disability onset and duration vary among individuals and with genotype, and reversible physical state transitions have been observed [8]. However, different categories of late-life disabilities have so far not been described in worms.

Human health-span is the duration of being in good health. Health, however, is a continuous state with different levels of robustness and difficult to define [21]. By contrast disability can be measured by self-reporting of difficulties in performing basic daily living tasks and by standardized tests of physical ability. In laboratory flies, climbing the wall of the food vial is one of the most abundant daily living activities. Accordingly, the ability to climb and to perform negative geotaxis is commonly used to measure health status [30,43]. We found that climbing disability is intimately associated with natural death, because flies develop this condition few days before death. Therefore, we used the disability to climb as a functional threshold measure. This allowed us to define health-span in flies with an accuracy of 6-24 hours at the individual level. In flies, death follows within few days upon the transition from heath- to ill-span. Similarly, physical disability is the best predictor of all-cause mortality and goes along with locomotor speed decay [8,42]. However, in humans gradual locomotor speed decay is observed decades before difficulties in performing basic daily activities occur. Similarly, in flies locomotor speed decreases several weeks before climbing disability.

### Life prolonging dietary interventions and late-life quality

Animal models are commonly used to test life-extending manipulations. We utilized our analysis of *Drosophila* late-life demography to evaluate effects of life-prolonging dietary interventions on late-life quality. Lifespan can be extended by feeding flies with increased carbohydrate/protein ratio diets [17,44]. We found that high sugar diet significantly increased lifespan and late-life quality scores in both genders, thus increasing health-span and compressing morbidity. It has previously been shown that high carbohydrate to protein diet increases lifespan [15,16]. Moreover, longevity requires both, high carbohydrate and low protein consumption [17]. However, because it has been reported that food consumption is best matched to a constant daily caloric intake target, increased concentration of sugar within the diet does not necessarily result in increased sugar consumption [17]. Therefore, at present, it remains unknown whether the beneficial effects of high carb/protein diet on late-life quality are a consequence of a shifted ratio of sugar to protein consumption, or alternatively, of dietary restriction. Please note that at a given caloric intake, high sugar concentration in the diet has been reported to promote obesity during aging [45], which would likely decrease late-life quality. Therefore, without further measurements of food intake and consumption a deeper discussion on the underlying mechanisms remains speculative.

We also tested the dietary supplements curcumin and super fruits (SF). Curcumin is a component of the spice turmeric, has antioxidant, anti-inflammatory, anti-mutagenic, anti-microbial, and neuroprotective effects [44,46,47], and it prolongs lifespan [22]. Super Fruit, a mix of açai, goji, pomegranate, mangosteen, noni and other extracts, protects flies against oxidative stress [23]. We confirmed that both antioxidants increased lifespan, but we detected variable effects on late-life quality, depending on the sugar content of the base diet and on gender. SF had no beneficial effect on male late-life quality, but diminished the positive effects of high sugar diet on female late-life quality. Curcumin had negative effects on the life quality of elderly females, and it significantly decreased beneficial effects of high sugar diet on elderly males. Indeed the combination of high sugar diet and curcumin led to a distinct pathological phenotype with signs of sugar mishandling and increased disability. It is possible that larval food supplementation with curcumin decreases the production of endogenous anti-oxidants in a compensatory manner, which turn, then renders adult flies more susceptible to oxidative stress, which is a known hallmark of aging. In fact, gene expression data from curcumin fed larvae shows inhibition of the TOR pathway in adults. This in turn increases lifespan, but in parallel accelerates leg neuromuscular degeneration [22].

Although, the underlying mechanisms require further analysis, our data show that pre-death disability can be compressed by dietary manipulation. It further demonstrates usefulness of the *Drosophila* model to evaluate the effects of life prolonging manipulation on late-life quality. Although the *Drosophila* system does certainly not recapitulate all aspects of mammalian late-life pathophysiology, together with the short generation times and versatile genetics, our analysis of *Drosophila* late-life demography can now be used to uncover molecular and cellular underpinnings of healthy aging.

## STATEMENT

Although *Drosophila* has been used widely for aging research, the question of how individual flies age with respect to their general health and motor performance remains largely unknown. In this study we provide longitudinal mapping of age-dependent motor decline in wild type *Drosophila* with high temporal resolution in multiple cohorts of flies (in total > 1000 individual flies). Our analysis unexpectedly reveals that flies age in one of two ways; one involving a gradual attrition of normal motor function culminating in death, and two, prolonged fitness until the end of life preceded by a short duration of hyperactivity. We next demonstrate usefulness of our analysis for evaluating the effects of life prolonging dietary intervention on health-span and late-life quality

## MATERIALS AND METHODS

### Animals

Oregon-R-C wild type flies (Oregon-R, strain: # 5, Bloomington fly stock center) were reared in cylindrical plastic vials (30 x 85 mm) at 25-27 ^o^ C and >65% relative humidity under a 12 h on–off light cycle. In order to rule out potential biases in our data, we confirmed that overall lifespan distributions and deficits are consistent in three experimental runs (Supplementary Figure 1). Each run was performed with a different batch Oregon-R flies (Bloomington fly stock center). For each run, from an expanded population (10 vials X ~50 flies) freshly emerged flies were collected daily, kept in groups (~20 animals of both sexes/vial), and transferred to fresh medium every 2–3 days. Males were scored for survival. Trays of vials were placed at random positions into the incubator and positions were rotated after each transfer to minimize the effects of microclimate. Any animal that had a non age-related death (e.g., stuck in the food, killed or lost during transfer) was censored. Male flies that reached the age of 59 days post adult eclosion were singled out, each in a separate food containing vial. On day 60 each fly was pre-examined for deficits (according to its behavioral responses to mechanical stimuli, see below), and only healthy individuals entered the longitudinal study. Singling out and longitudinal testing did not affect the survival rates as compared to control cohort (see Supplementary Figure 1, Figure 4A). Longitudinal testing was conducted either by testing each individual fly in the startle (see below) assay every 6 hours from day 60 until death, or by continuous video observation between day 60 and death. Vials were replaced every second day until an individual was called dead (see text for death criteria). Diet was based on corn flour-yeast-agar medium containing 0.83% (w/v) agar, 1.3% (w/v) semolina, 3% (w/v) corn meal, 0.25% (w/v) soybean meal, 3.5% (w/v) dry yeast, 0.7% (w/v) fructose, 3.3% (w/v) sucrose, 0.1 (w/v) CaCl_2_, 2.5% (v/v) nipagen diluted in 10% absolute ethanol, 0.42 (v/v) propionic acid.

### Dietary supplements and feeding protocol

Curcumin was obtained from Sigma >95% pure (#28260). Super fruits (açai, goji, pomegranate, mangosteen, noni, vegetable cellulose, microcrystalline cellulose, natural colors, stearic acid, magnesium stearate, silica, and purified water) was obtained from Nature's Plus. Curcumin (100 mM [40],) or Super Fruit (50 mg/ml [23],) were incorporated into the food by direct vigorous mixing of the chemical with food, following the instructions of a previous study [22]. This mixing approach was proven to be effective since larvae and early adults fed on fly food supplemented with curcumin live longer [22]). To avoid degradation of antioxidants, the food was cooked without SF and curcumin, then cooled down to 35°C to add the supplements. The basic (Standard) media with carbohydrate/protein ratio 2/1 contained 0.75% (w/v) agar, 4.5% (w/v) dry yeast, 3.5% (w/v) corn meal, 5.5% (w/v) Sucrose, 0.4% (v/v) Propionic acid, 2.5% (v/v) nipagen diluted in 10% absolute ethanol. Standard media with carbohydrate/protein ratio 8/1 contained the same concentrations of ingredients with quadruple Sucrose concentration (22% w/v). The basic media (carbohydrate/protein ratio 2/1) was also used for feeding Lausanne-S (strain # 4268, Bloomington fly stock center).

For the dietary run, we followed the protocol outlined in Figure 6E. From an expanded Oregon-R population (20 vials X ~50 flies) freshly emerged flies of both sexes were collected daily for a period of five days and transferred to fresh basic medium (c:p 2:1) alone, or to basic medium (c:p 2:1) supplemented with curcumin, or to basic medium (c:p 2:1) supplemented with Super Fruit (Figure 6E). A day later parents were removed and larvae as well the emerged adults were fed with the one of the three above mentioned diets. Three days old adults were then divided into two groups, the first group was continuously fed on basic media c:p 2:1 diet. The second group was transferred to a basic media c:p 8:1 diet. A the age of 60 days males and females of the first and second group were singled out in vials containing the same basic media (c:p 2:1) and (c:p 8:1) correspondingly (Figure 6E).

### Behavioral assays

### *Startle assay*

The Zeitgeber was a 12h light/dark cycle with light-on at 8am and light-off at 8pm. Experimental testing was done every 6 hours at 7am (dark), 1pm (light), 7pm (light), and 1 am (dark). Experimental testing was done under red light to not interfere with the light/dark cycle of the flies. Escape performance was tested in individual flies in food vials by gently banging the vial on the counter. The physical status of the individual was defined by its motor responses to this mechanical stimulation. The assay was personalized to address heterogeneity in behavioural performance, because even at identical ages, individuals exhibited a broad variability in physical status, vigour and arousal. Five sequences of testing with one minute rest in between each sequence were conducted (each sequence consisted of 3-5 gentle but abrupt tapings of the vial onto the counter for a duration of approximately 1 s each). For impaired individuals (flies that failed to respond to all 5 mechanical stimulation sequences), single light tapings were used to elicit walking responses, thus allowing to diagnose walking impairments. The criteria used to evaluate the physiological status of the flies were pre-evaluated and defined in two preliminary longitudinal studies (N=30 males, age >60 days old, tested daily, N=25, age >65 days old, tested every 3 hours) and by numerous observations of age-related pathological signs in individuals from different laboratory stains. The resulting physiological status criteria are listed below and were assigned with scores (see Table 1): fit (scores 1-3), the fly is fully capable to escape by performing climbing (1), jump (2), or flight (3) in response to at least one of the five stimulation sequences); mild climbing impairment (-1), the fly has acquired an obvious climbing deficit (e.g. leg dragging) but is still capable to fully climb the wall of the vial in response to at least one of the 5 subsequently delivered mechanical stimuli; moderate climbing impairment (-2), in its best response performance the fly climbs no higher than the 1/3 of the vial’s height; severe climbing impairment (-3), the fly can barely climb (few mm) and regularly falls off the wall of the vial; complete climbing impairment (-4), the fly fails to climb in response to any of the 5 stimuli; paradoxical behavior (-1), the fly performs unexpected responses to the stimulation (e.g. walking in circles, aggressive behavior against no opponent, coma-like state lasting for seconds to minutes followed by full recovery, spontaneous seizure-like behavior); responsiveness impairment, the fly exhibits a delayed, low intensity response (few steps only) or no response can be observed; terminal stage (-5), the fly is moribund and in a death posture (mostly on its back, supine).

### Video recordings

Individual > 60 days old male flies reared in food vials or in custom made plexiglass devices (4 x 4 x 0.5cm) were continuously filmed (1920x1080 pixels) under red light with a Sony digital video camera (Sony Handycam HDR-SR11E, Sony Inc.) until death. The last 11 hours of life were analyzed in bins of 10 minutes. The following parameters were measured: the percentage of time walking at any place in the vial; the number of successful or unsuccessful efforts to climb the wall of the vial; the time spend by an individual at the wall or the bottom of the vial; feeding times; the duration and number of events indicating pathological signs. These included the time spend on the wall or the bottom of the vial, feeding times, the duration and number of events of posture and walking disabilities, righting behavior, seizure-like behavior, leg shaking, rhythmical proboscis extraction/retraction and abdominal bending, supine position, and terminal stage.

### Electrophysiology

Leg motoneuron stimulation and muscle action potential recording were performed with tungsten electrodes. Recorded signals were amplified 1000-fold with an AC Amplifier (AM systems), digitized without filtering (digidata 1200, Axon Instruments) and analyzed with pClamp software. A Grass Stimulator was used for extracellular stimulation.

### Statistical analysis

Statistical analysis was performed using GraphPad Prism version 6.00 for Windows (GraphPad Software, La Jolla California USA, www.graphpad.com) and SPSS statistics. All data were first tested for normality (D’Agostino &Pearson omnibus normality test). Normally distributed data were presented as means and SEM. Students T-test was used to test for statistically significant differences between two groups. ANOVA with Tukey and/or LSD posthoc testing was used for statistical comparison of more than two test groups. If normality did not hold, non-parametric analysis was used accordingly. Mann-Whitney U-test for comparison between 2 groups and Kruskal-Wallis ANOVA with Dunn's posthoc testing for comparison among more than 2 groups. Significance levels were defined as *p < 0.05; ** p < 0.01, ***p < 0.001).

### *Principal component analysis*

To determine if the data extracted from continuous motor activity measurements during the last 11 hours prior to death contained quantitatively different traits, we grouped the animals as either having a “sudden death” or a “silent death” phenotype according to their climbing activity. All tests described below were done using the “R” statistical package (https://www.r-project.org/). We first tested if there was reason to believe that one group was inherently larger than the other by employing the Binomial test for proportions, specifically, a 1-sample proportions test without continuity correction. Finding no reason to accept this hypothesis, we used the Kolmogorov-Smirnoff nonparametric comparison of two distributions (D = 1, p= 1.604 X 10^-05^). This verified our hypothesis that there are two distributions. As there are ties in the data, exact p-values could not be computed so the test was run a second time, with a bootstrap (nboots = 10000) yielding the same results. The uncorrected Welch two-sample t-test showed that the difference in means between the two populations was significant for the untransformed data (t = -7.3009, df = 7.688, p = 0.0001024; Mean for silent death = 35.27273, suddent = 341.12500) and also for the transformed data by taking the square root (t = -9.8127, df = 16.234, p = 3.126e-08; Mean for silent death = 4.398886, Mean for sudden death = 18.235005). Cluster analysis was done to extract an objective breakpoint between the two groups, which yielded a value of a total of 150 climbing efforts / 700 min and produced scree and cluster plots showing that two groups explain 100% the variability.

## Supplementary Material

Supplementary Figures

Movie 1

Movie 2

Movie 3
