# Supplementary material for: Longitudinal assessment of health-span and pre-death morbidity in wild type Drosophila
Source: Aging (Albany NY). 2019 Mar 27;11(6):1850–73. doi: 10.18632/aging.101880 (PMC6461171; doi:10.18632/aging.101880)
Supplement: Supplementary Figures [file aging-11-101880-s001.pdf]

SUPPLEMENTARY FIGURES

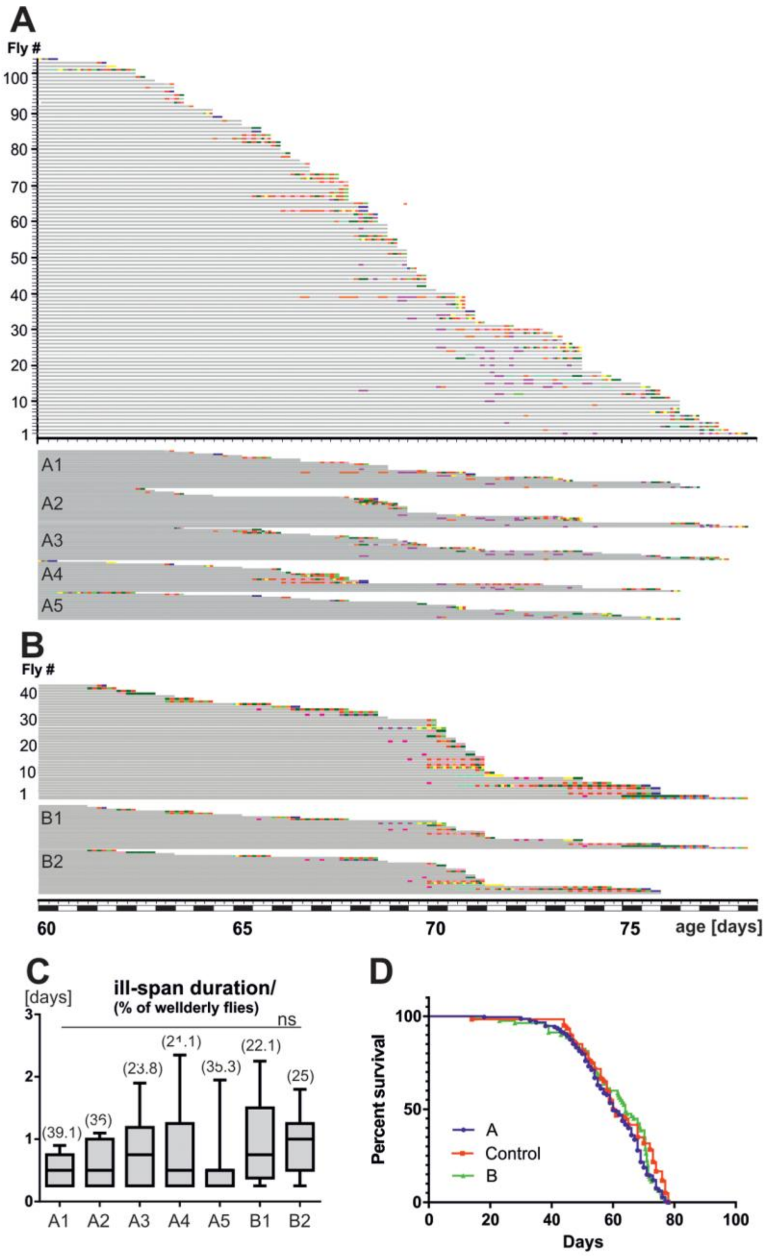

Supplementary Figure 1. Two experimental runs reveal no differences in late life pathology. (**A**) Life history chart of the 104 Oregon-R male flies shown in Figure 1A. **(A1-A5)** Five replicates of the cohort shown in **A**. (**B**) Life history chart of 48 male flies (2 replicates, **B1**, **B2**) individually tested in the startle assay every 6 hours from the age of 60 days until death. These flies derived from a different Oregon-R batch. As in Fig. 1A, B, C gray bars indicate health-span and colored bars different disability categories (see colored inset in Fig. 1A). Black and white bars on x-axis indicate day-night cycle. (**C**) The duration of ill-span is not statistically significant between groups (Kruskal-Wallis test p= 0.4425, Dunn's multiple comparisons posthoc test, p > 0.9999 for all pairs). Numbers in parenthesis above bars indicate percentage of wellderly flies. (**D**) The survival curves of tested populations in A and in B and in the non-tested control flies are not significantly different (Logrank test for trend; X^2=3.110, 1 df, p=0.0778).

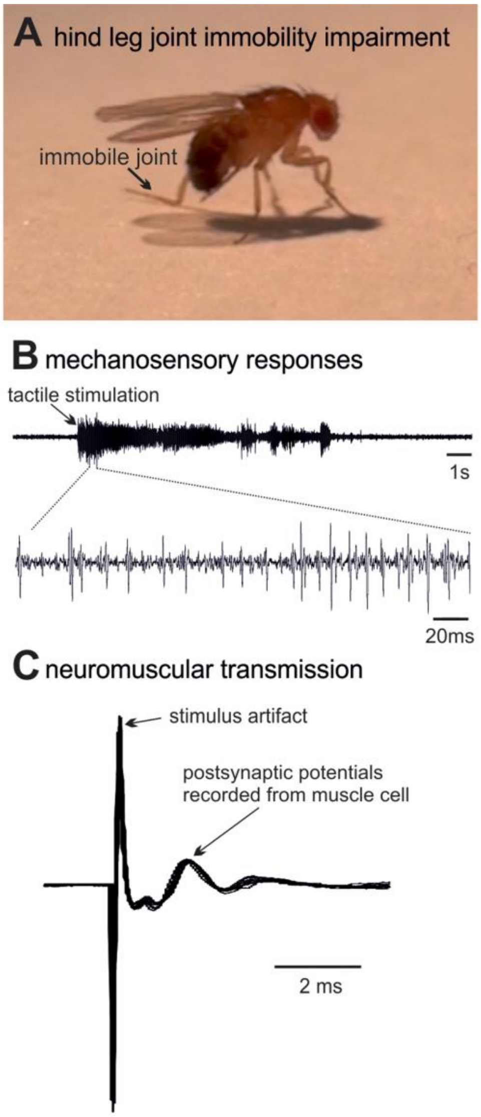

Supplementary Figure 2. Sensory and muscle cells respond to stimulation in immobile legs. (**A**) Photograph of a fly with a representative immobility impairment of the left hind leg (arrow) that caused leg dragging during walking. (**B**) Extracellular recording of sensory nerve activity in response to a tactile stimulation at the tibia. Arrow indicates time point of tactile stimulation. (**C**) Overlays of 10 sweeps of muscle potentials recorded extracellularly from the flexor tibia muscle in response to electrical stimulation of the leg motoneurons with a sharpened tungsten electrode.

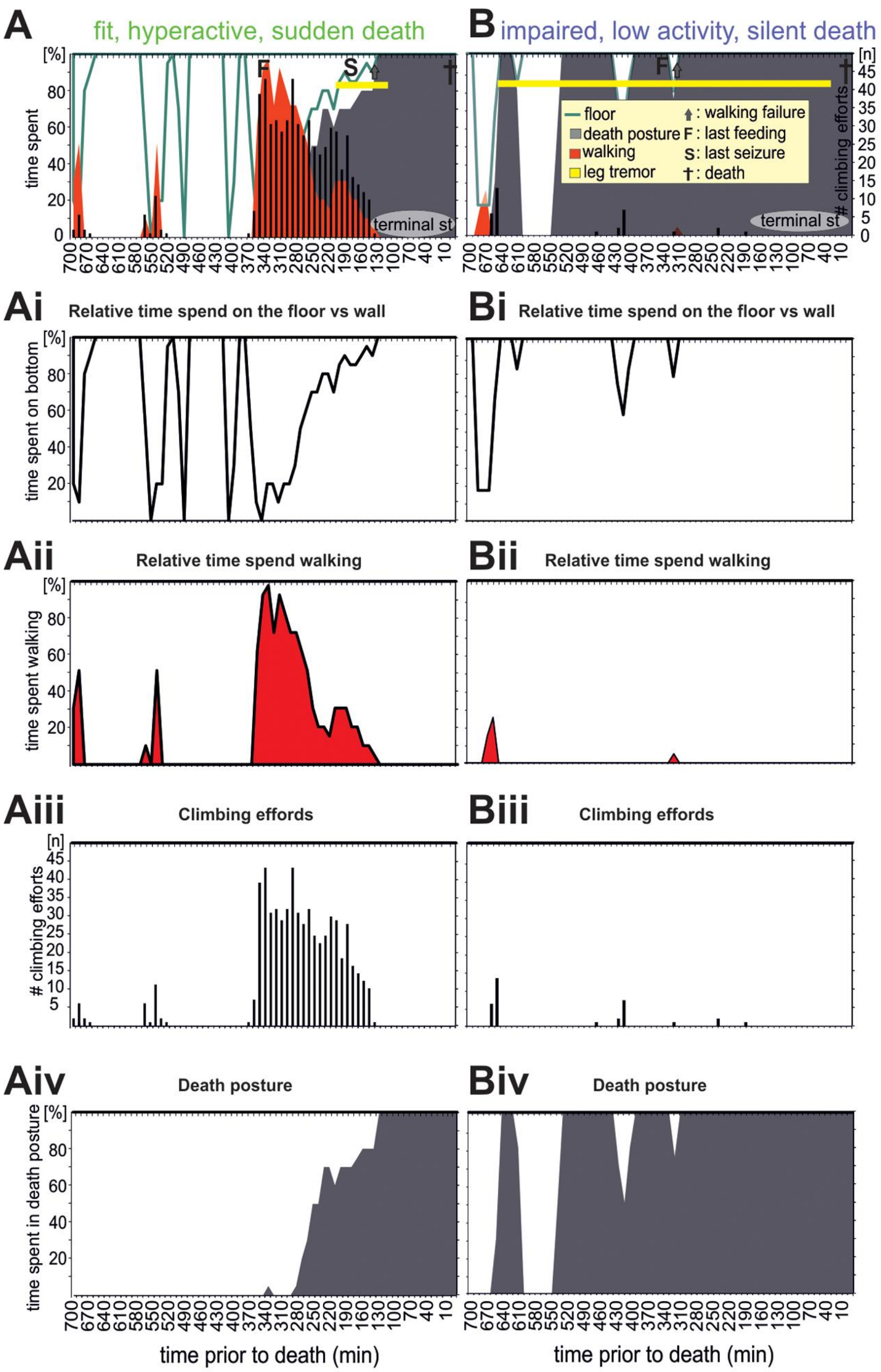

Supplementary Figure 3. Two types of pre-death pathophysiology. Analysis of figure 4D, E. (**A, B** Representative animal with high locomotor activity and no impairments until the last day (**A,** sudden death) as compared to a representative animal with impairments and low locomotor activity (**B,** silent death).The occurrence of different spontaneous behaviors during the last 11 hours of life plotted as cohort averages in time bins of 10 minutes (x- axis). Percent time spent standing on the ground of the vial (**Ai, Bi**), walking (**Aii, Bii**), the number of climbing efforts (**Aiii, Biii**) and being immobile or in a supine position (**Aiv, Biv**).
